# Effect of undigestible neutral detergent fiber concentration in finishing diets containing dry-rolled or steam-rolled barley for feedlot steers

**DOI:** 10.1093/jas/skae392

**Published:** 2025-04-08

**Authors:** Stephanie A Terry, Wenzhu Yang, Karen A Beauchemin, Karen S Schwartzkopf-Genswein, Gregory B Penner, Katharine M Wood, Tim A McAllister

**Affiliations:** Agriculture and Agri-Food Canada, Lethbridge Research and Development Centre, Lethbridge, AB, Canada T1J 4B1; Agriculture and Agri-Food Canada, Lethbridge Research and Development Centre, Lethbridge, AB, Canada T1J 4B1; Agriculture and Agri-Food Canada, Lethbridge Research and Development Centre, Lethbridge, AB, Canada T1J 4B1; Agriculture and Agri-Food Canada, Lethbridge Research and Development Centre, Lethbridge, AB, Canada T1J 4B1; Department of Animal and Poultry Science, University of Saskatchewan, Saskatoon, SK, Canada S7N 5A8; Department of Animal Biosciences, Ontario Agricultural College, University of Guelph, Guelph, ON, Canada N1G 2W1; Agriculture and Agri-Food Canada, Lethbridge Research and Development Centre, Lethbridge, AB, Canada T1J 4B1

**Keywords:** cattle, feeding behavior, fiber, grain processing, rumen, uNDF

## Abstract

This study evaluated the impact of grain processing method (dry- vs. steam-rolling) and diet uNDF concentration (low vs. high; 5.7% vs. 6.6% on DM basis by replacing silage with straw) in finishing diets on feed intake, feeding behavior, growth performance, ruminal pH, fermentation parameters, inflammatory stress responses, and carcass traits of 376 intact (initial body weight (BW) ± SD, 440 ± 33.6 kg), and 24 ruminally cannulated steers (initial BW ± SD, 474 ± 30.5 kg). Steers were housed in 32 pens with 4 pens of 30 steers, and 28 pens of 10 steers. Eight of the pens with 10 steers, and all of the pens with 30 steers were equipped with GrowSafe to record individual feed intake and feeding behavior. Three cannulated and 7 intact steers were housed in the smaller GrowSafe pens. Diets included (1) dry-rolled barley and barley silage; (2) dry-rolled barley and barley straw; (3) steam-rolled barley and barley silage; and (4) steam-rolled barley and barley straw, formulated to contain 89% barley-grain, 6% roughage and 5% vitamin and mineral supplement (DM basis). Interactions between the processing method and uNDF concentration were observed for maximum ruminal pH, and concentrations of blood glucose and lipopolysaccharide-binding proteins. Compared to dry-rolling, steam-rolling did not improve growth performance, ruminal pH, fermentation parameters, or liver abscess scores, but did increase longissimus muscle (**LM**) area (*P* = 0.01) and decrease the proportion of AAA carcasses (*P* = 0.01). Steam-rolled barley decreased (*P* = 0.04) glucose and increased (*P* = 0.01) blood concentrations of insulin and acute phase proteins. Increasing uNDF did not affect feed intake, growth, carcass traits, or liver abscess scores, but did increase (*P* = 0.01) bunk attendance, meal duration, and to a lesser extent meal intervals (*P* = 0.04) and eating rate (*P* = 0.01). Increased uNDF raised (*P* = 0.04) mean ruminal pH and reduced the duration of pH below 6.0, 5.8, and 5.2, and tended (*P* = 0.06) to increase the acetate to propionate ratio. The lack of growth response to dietary uNDF concentration could be due to the small differences in uNDF intake, or that uNDF concentration was sufficient to prevent digestive disturbances. Increasing dietary uNDF altered eating behavior and ruminal pH in a manner that could reduce the risk of clinical and subclinical ruminal acidosis.

## Teaser

Increasing undigestable neutral detergent fiber concentration while keeping the forage concentration constant in high-grain finishing diets reduces ruminal acidosis and maintains growth performance of feedlot cattle.

## Introduction

In North American feedlots, it is common practice to incorporate high-energy grains and minimize forage in finishing diets to achieve maximum growth performance, feed conversion, and profits as the energy value and digestibility (Mertens, 1997; Galyean and Defoor, 2003) of grains is usually greater than forages (Mertens, 2002). The disadvantage of this practice is that feeding rapidly fermentable grain increases the risk of ruminal acidosis and subsequent metabolic disorders such as abscessed livers, rumenitis, and lameness (Nagaraja and Lechtenberg, 2007; NASEM, 2016). These disorders are associated with a decrease in productivity, health, welfare, and profitability (Pereira et al., 2020; Ran et al., 2021). Although minimum forage inclusion rates are often used in finishing diets, there are no strict guidelines for minimal forage requirements for finishing beef cattle. A number of factors likely affect the minimal forage requirement, including forage particle size and digestibility, grain type and processing, and concentration of lipids in the diet (Koenig and Beauchemin, 2011). Considering that dietary fiber is a key mitigant of ruminal acidosis, different aspects of fiber, including neutral detergent fiber (**NDF**), effective NDF (Mertens, 1997), physically effective fiber (**peNDF**), and undigestible NDF (**uNDF**) have been assessed for their role in reducing the risk of ruminal acidosis in cattle-fed high-grain diets (Reinhardt and Hubbert, 2015).

Physically effective NDF considers physical characteristics (particle size) and NDF concentration of forages that are known to promote rumination and formation of the ruminal mat (Mertens, 1997). Increasing peNDF in beef cattle diets increases the time spent eating and ruminating (Ran et al., 2021) and saliva production. Greater concentrations of peNDF can result in increased ruminal pH (Weiss et al., 2017), digestibility (Yang and Beauchemin, 2005), growth (Gentry et al., 2016), and improved carcass traits (Pereira et al., 2021). Although the peNDF system represents a major advancement in characterizing the impact of physical aspects of NDF on rumen function, its use with feedlot diets has been limited (National Research Council, 2001; NASEM, 2016; Samuelson et al., 2016). Interpretation of the value of peNDF in high-concentrate feedlot diets is also impacted by whether estimates are based on the complete diet or just the forage or byproduct portion of the diet (Pereira et al., 2023a). In particular, it does not adequately account for the digestibility of NDF or non-structural carbohydrates, nor for effects on volatile fatty acid (**VFA**) production and ruminal pH (White et al., 2017).

Undigestible NDF has also been used to describe dietary fiber, and is receiving increased interest for its potential effects on ruminal physical fill and motility; factors that may impact feed intake and VFA absorption. Recent studies have shown that the inclusion of uNDF improved the prediction of DM intake (DMI) in dairy cattle-fed forage-based diets (Grant et al., 2020). It was linked to altered eating behavior (Pereira et al., 2021, 2023b; Ran et al., 2021), ruminal motility (Pereira et al., 2023a), and pH (Ran et al., 2021). Sá Neto et al. (2014) reported that increasing diet uNDF (10.5% to 13.0% of dietary DM) by replacing corn silage with sugarcane silage in a dairy cattle diet, increased chewing time (from 116 to 137 min/kg forage NDF), a response that was not reflected by changes in peNDF. Consequently, peNDF on its own may not be as effective as uNDF at considering impacts on rumination and ruminal function (Grant et al., 2020). Ran et al. (2021) reported that increasing the concentration of uNDF (4.6% vs. 5.6% of dietary DM) in the diet of finishing beef heifers resulted in longer meal duration and chewing times. Pereira et al. (2023a) concluded that the combination of peNDF and uNDF was a more meaningful method of characterizing the functionality of fiber in finishing feedlot cattle diets.

The objectives of this study were to investigate effects of barley processing method (dry-rolled vs. steam-rolled) and concentration of uNDF inclusion in finishing diets on feed intake, eating behavior, growth performance, ruminal pH and fermentation characteristics, inflammatory and stress responses, and carcass traits of feedlot steers. We hypothesized that increasing uNDF in steam-rolled barley would have a positive impact on ruminal pH, and would thereby improve growth performance and reduce indicators of systemic inflammation as compared to dry-rolled barley.

## Materials and Methods

This study was conducted at the Feedlot Facility of Agriculture and Agri-Food Canada, Lethbridge Research and Development Centre (Lethbridge, AB, Canada) in accordance with guidelines of the Canadian Council on Animal Care (2009) and was approved (protocol #ACC2017) by the Institutional Animal Care and Use Committee.

### Animals, treatments, and experimental design

Four hundred Angus beef steers (initial body weight [**BW**] ± SD = 440 ± 33.6 kg), of which 24 were ruminally cannulated (initial BW ± SD = 474 ± 30.5 kg), were housed in 32 feedlot pens. Four large pens housed 30 steers, and 28 small pens housed 10 steers each. The large pens and 8 of the small pens were equipped with a feed bunk monitoring system (GrowSafe Systems Ltd., Airdrie, AB, Canada) for continuous recording of individual animal feed intake and eating behavior. Three cannulated and 7 intact steers were assigned to each of the small pens equipped with GrowSafe bunks. All steers were provided with free access to fresh water via an automatic waterer shared between 2 pens.

Upon arrival at the feedlot, steers were vaccinated with Express Yearling (Ingelheim Ltd., Burlington, ON, Canada) and Tasvax 8 (Schering-Plough Animal Health Ltd., Upper Hutt, New Zealand). Steers were treated with the parasiticide, Bimectin (5 mg of ivermectin/mL) pour-on (Bimeda MTC Animal Health Inc., Cambridge, ON, Canada), and implanted with Component TE-S with tylosin (Elanco Division of Eli Lilly Canada Inc., Guelph, ON, Canada). Each implant contained 120 mg trenbolone acetate, 24 mg estradiol UPS, and 29 mg tylosin tartrate.

The experiment was conducted as a 2 × 2 factorial arrangement of treatments, with 2 grain processing methods (dry-rolled and steam-rolled) and 2 concentrations of uNDF (low and high uNDF). The uNDF concentration in the diet was adjusted by replacing barley silage with straw. This resulted in 4 dietary treatments: 1) dry-rolled barley and barley silage (Low uNDF); 2) dry-rolled barley and barley straw (High uNDF); 3) steam-rolled barley and barley silage (Low uNDF); and 4) steam-rolled barley and barley straw (high uNDF). Diets were formulated with 89% barley-grain, 6% roughage and 5% vitamin and mineral supplement (Table 1, DM basis) to meet or exceed requirements of finishing feedlot steers (NASEM, 2016). Steers were transitioned to high-grain diets over 15 d using 3 step-up diets where the concentrate concentration was increased by an equal proportion in each diet so as to reach the final concentration in the finishing diet. Diets were fed once daily as a total mixed ration (**TMR**) using slick bunk management practices and all contained monensin at 33 mg/kg DM (Rumensin Premix, Elanco Health, Mississauga, ON, Canada). Treatments were evenly distributed for each pen type, with 8 pens assigned to each treatment (1 large pen and 2 small pens with GrowSafe, and 5 small pens without GrowSafe). Cattle were fed the finishing diet for 112 d after which they were marketed at a liveweight between 650 and 750 kg.

**Table 1.** Ingredients and chemical composition of diets

	Dry-rolled		Steam-rolled	
	Low^1^	High^1^	Low	High
Ingredients, % of DM				
Barley silage	6	—	6	—
Barley straw	—	6	—	6
Barley-grain, dry-rolled	89	89	—	—
Barley-grain, steam-rolled	—	—	89	89
Supplement^2^	5	5	5	5
Nutrient content, % of DM				
DM, %	85.0 ± 1.27	92.0 ± 2.78	84.1 ± 1.37	90.9 ± 1.23
OM	91.2 ± 0.26	91.2 ± 0.29	91.0 ± 0.30	91.1 ± 0.27
NDF	16.2 ± 1.98	18.0 ± 1.21	16.7 ± 1.44	18.4 ± 1.94
uNDF^3^	5.5	6.4	5.9	6.8
ADF	5.6 ± 0.64	6.7 ± 1.20	5.9 ± 1.33	7.0 ± 0.76
Starch	49.3 ± 4.30	48.2 ± 1.95	51.4 ± 9.64	50.3 ± 2.43
CP	12.9 ± 0.13	12.2 ± 0.41	12.3 ± 0.97	11.7 ± 0.88
peNDF of TMR				
Pef^4^	0.052	0.046	0.666	0.659
peNDF^5^, % of DM	0.84	0.83	11.43	12.42
peNDF of forage				
Pef^4^	0.051	0.044	0.051	0.044
peNDF^5^, % of DM	2.35	3.34	2.35	3.34

^1^Low = low concentration of undigestible NDF (uNDF); high = high concentration of uNDF.

^2^Composition: 54.7% ground barley, 9.7% canola meal, 24.3% calcium carbonate, 2.3% molasses, 5% salt, 2% urea, 0.07% vitamin E (500,000 IU/kg) and beef feedlot premix (supplied: 15 mg Cu, 65 mg Zn, 28 mg Mn, 0.7 mg I, 0.2 mg Co, 0.3 mg Se, 6,000 IU vitamin A, 600 IU vitamin D, and 47 IU vitamin E per kilogram of dietary DM).

^3^uNDF, undigestible NDF, measured in situ after 240 h of incubation in rumen. Calculated from individual ingredient composition.

^4^Physical effectiveness factor determined as the sum of particles retained on 19- 8- and 4-mm sieves. Calculated from individual ingredient composition.

^5^Physically effective NDF estimated as the NDF concentration of feeds multiplied by its pef. Calculated from individual ingredient composition.

### Grain processing, and peNDF and uNDF determination

Barley-grain from the same lot was either dry- or steam-rolled. Steam-rolled barley was conditioned for 20 min at 104 °C (set point) and rolled to a density of 465 g/L. Barley in both treatments was processed to a processing index (**PI**) of 70 % (PI = density after processing/density before processing × 100). The selected PI was based on previous experiments (Nixdorff et al., 2020; Ran et al., 2021) that suggested alterations in uNDF would impact rumen function when forage constituted 6% or less of diet DM. To determine particle size distribution, processed barley was sieved through a series of screens (4.0, 3.35, 2.36, 1.18, and 0.85 mm) using a Ro-Tap Sieve shaker (Ro-Tap Sieve Shaker; Laval Lab Inc., Laval, QC, Canada).

The NDF, peNDF, and uNDF content of barley silage, barley straw, dry-, and steam-rolled barley were determined. The concentration of uNDF was determined in situ using 3 ruminally cannulated beef heifers fed a high-forage diet containing 60% barley silage, 30% barley straw, and 10% protein, mineral, and vitamin supplement (DM basis; Beauchemin et al., 2019). Briefly, 4 samples of each feedstuff collected at equal intervals throughout the experiment were dried at 55 °C for 48 h (Terry et al., 2023), or until a stable weight was reached. Barley straw and silage were ground through a 2-mm screen, and the barley-grain was ground to pass a 4-mm screen using a Wiley mill (standard model 4, Arthur H. Thomas Co., Philadelphia, PA). Substrates were weighed into 10 × 20 cm polyester bags (R1020, ANKOM Technology, Macedon, NY; 50-μm porosity) with approximately 10 ± 0.05 g of barley straw and silage DM, and 20 ± 0.05 g of barley-grain DM per bag. For each heifer, triplicate bags of each substrate were placed inside large mesh bags (30 × 30 cm) and incubated in the rumen for 240 h. Upon removal from the rumen, bags were immediately submerged in ice water to impede microbial activity and washed under cold running water until the water ran clear. Excess water was removed by gentle squeezing and the bags were dried in a forced-air oven at 55 °C for 72 h. Remaining residue in each bag was ground to pass through a 1-mm screen and analyzed for NDF in a fiber analyzer (F57 Fiber Filter Bags, 200 Fiber Analyzer, ANKOM Technology, Macedon, NY) using sodium sulfite and heat-stable amylase. The uNDF concentration was calculated as the amount of NDF remaining in the residue after incubation, expressed as percentage of NDF before incubation. Physical effectiveness factor (pef) was determined as the collective proportion of feed particles (as fed basis) retained on the 19-, 8-, and 4-mm sieves using a Penn State Particle Separator (Nasco, Ajax, ON, Canada). The peNDF based on TMR or only forage fraction was calculated with pef multiplied by its NDF content (Weiss et al., 2017; Tables 1 and 2).

**Table 2.** Chemical composition and particle size distribution of barley-silage, barley-straw and rolled barley-grain

	Barley silage	Barley straw		Dry-rolled barley	Steam-rolled barley
Nutrients, % of DM					
DM, %	36.9 ± 3.42	92.0 ± 1.79		94.1 ± 0.89	91.1 ± 1.01
OM	90.3 ± 1.28	91.8 ± 0.71		96.3 ± 0.24	96.1 ± 0.60
NDF	46.5 ± 0.78	76.0 ± 1.23		15.1 ± 0.51	15.6 ± 0.04
uNDF^1^	14.5 ± 1.16	26.8 ± 1.02		5.2 ± 0.40	5.7 ± 0.26
pdNDF^2^	32.5 ± 1.16	49.3 ± 1.01		9.9 ± 0.40	9.9 ± 0.26
ADF	25.2 ± 0.12	43.6 ± 1.19		4.6 ± 0.01	4.9 ± 0.13
Starch	19.3 ± 1.25	1.7 ± 0.48		54.1 ± 2.15	56.4 ± 0.30
CP	12.39 ± 0.05	4.90 ± 0.05		13.72 ± 0.07	13.46 ± 0.02
Particle size distribution, %					
PSPS^3^			Ro-Tap Sieve shaker^4^		
>19 mm	2.2 ± 0.07	13.0 ± 0.09	>4.0 mm	0.2 ± 0.02	65.5 ± 0.82
8 mm	32.0 ± 0.59	37.1 ± 0.45	3.35 mm	6.8 ± 0.10	25.6 ± 0.58
4 mm	50.2 ± 0.45	23.3 ± 0.16	2.36 mm	36.6 ± 0.12	5.2 ± 0.13
<4 mm	15.6 ± 0.06	26.6 ± 0.20	1.18 mm	46.4 ± 0.18	1.6 ± 0.09
			0.85 mm	3.9 ± 0.09	1.0 ± 0.06
			<0.85 mm	6.1 ± 0.02	1.1 ± 0.01
pef^5^	0.844 ± 0.001	0.734 ± 0.002		0.002 ± 0.0002	0.655 ± 0.008
peNDF^6^	39.21 ± 0.03	55.76 ± 0.16		0.03 ± 0.004	10.20 ± 0.12

^1^uNDF, undigestible NDF, measured in situ after 240 h of incubation in rumen.

^2^Potentially digestible NDF = NDF − uNDF.

^3^Penn State Particle Separator, % of sample retained on various screens.

^4^Ro-Tap Sieve shaker (Laval Lab Inc., Laval, QC, Canada); dry-rolled barley was processed to processing index of 70%; steam-rolled barley was rolled to a density of 450 g/L, % of sample retained on various screens.

^5^Physical effectiveness factor determined as the sum of particles retained on 19- 8- and 4-mm sieves.

^6^Physically effective NDF estimated as the NDF concentration of feeds multiplied by its pef.

### Growth performance and eating behavior

The amount of TMR offered was recorded daily for each pen to determine pen DMI, with feed refusals weighed and sampled weekly. Samples of TMR and pen feed refusals were sampled and dried weekly at 55 °C for 72 h for DM determination. Samples of dried TMR and ingredients were pooled monthly for further analysis. Pen DMI was calculated as the difference between TMR offered and weekly refusals, corrected for DM content. Only intact steers were included in growth performance and carcass characteristic measurements. Steers were weighed before feed delivery (non-fasted) on 2 consecutive days at the beginning and end of the experiment, and on 1 d each month. Average daily gain (**ADG**) was calculated by dividing weight gain by the number of days on feed. Feed efficiency (**G:F**) was calculated by dividing ADG by DMI.

Individual feed intake and eating behavior data were collected for a total of 200 steers over the duration of the trial using the GrowSafe system. Individual intake, frequency of visits, duration of total bunk attendance, eating rate (g DM/min), and average duration of each visit were recorded daily. Distinct feeding events were selected based on a meal criterion of 300 s as described by Schwartzkopf-Genswein et al. (2002). Individual eating data were summarized to report DMI, DMI variation (SD of intake over a week), meal frequency (number of meals/d), meal duration (min/meal), and inter-meal interval (min). Eating rate was determined as the total weight of all meals within a day divided by daily feeding time (g DM/min).

### Ruminal pH and fermentation characteristics

Approximately 500 g of ruminal digesta were collected from 24 cannulated steers at the start (day 0) of the trial, and monthly thereafter at weigh day. Contents of the reticulorumen were collected from the reticulum, and the ventral, caudal, and cranial sacs of the rumen, mixed, and squeezed through 2 layers of PECAP nylon (pore size 355 μm; Sefar Canada Inc., Ville St. Laurent, QC, Canada). Two samples of ruminal liquor (2 mL) were stored with 0.4 mL of 25% (wt/v) metaphosphoric acid (HPO_3_) and 0.4 mL of sulfuric acid (H_2_SO_4_) for analysis of VFA and NH_3_-N, respectively.

Following ruminal sampling, a pH logger (LRCpH measurement system, Dascor, Inc., Escondido, CA) was placed in the ventral sac of each cannulated steer for continuous measurement of pH every 5 min. Millivolt readings were used to standardize the loggers using pH 4 and 7 buffers and corrected for temperature. Loggers were placed in the rumen and pH was recorded through the third week of each month. The mV data obtained were converted to pH using beginning and ending linear regressions derived from the starting and ending standardizations with the assumption that drift was linear over the duration of measurement (Penner et al., 2006).

### Blood sampling and analysis

On days 0, 56, 112, all steers housed in the small GrowSafe pens were subjected to blood and hair sampling. Blood samples were collected from the jugular vein into 2, 10-mL non-additive tubes (BD vacutainer; Becton Dickinson Co., Franklin Lakes, NJ) for analysis of haptoglobin (**HP**), lipopolysaccharide-binding protein (**LBP**), serum amyloid-A (**SAA**), insulin, and glucose. Plasma samples were kept on ice, whereas serum samples were allowed to clot at room temperature before being centrifuged. All samples were centrifuged at 2,500 × *g* for 15 min at 4 °C, and stored at −20 °C until analysis. Hair samples were taken from the forehead and stored in plastic bags until analyzed for cortisol as an indicator of chronic stress (Moya et al., 2013).

Plasma samples were used to measure glucose and LBP, and serum was used to measure insulin, SAA, and Hp. For blood analyses, standard curves were included in each microplate and all microplates were read on an EPOCH2 Microplate Reader (BioTek Instruments, Inc., Winooski, Vermont). Plasma glucose was measured in triplicate using the glucose oxidase–peroxidase method (Sigma Aldrich, Oakville, ON, Canada) with an intra assay coefficient of variation (CV) of 1.71%. Serum insulin was measured in triplicate using a bovine-specific ELISA (Mercodia Inc., Winston-Salem, North Carolina) with an intra assay CV of 1.98 %. SAA was measured in triplicate using Tridelta Phase enzyme-linked immunosorbent assay (ELISA; Tridelta Development Limited, Maynooth, County Kildare, Ireland) with an intra assay CV of 4.37 %. Serum Hp was measured in triplicate with a sandwich ELISA (GenWay Biotech Inc., San Diego, CA), with an intra assay CV of 1.47%.

### Carcass characteristics and liver abscess scores

At the end of the trial, intact steers were slaughtered at Cargill Foods (High River, AB, Canada) and cannulated steers were processed at Alberta Prairie Meats (Duchess, AB, Canada). Hot carcass weight (HCW; with kidneys removed), dressing percentage, backfat thickness, longissimus muscle (LM) area, saleable meat yield, and quality grade of the intact steers were collected from the abattoirs visual (Canadian Beef Grading Agency) and Computer Vision Grading Systems (VBG 2000 e + v Technology GmbH, Oranienburg, Germany). Livers were graded using the Elanco Liver Check System (Elanco, 2019). Livers scores were classified as abscessed with at least 1 abscess, and severely abscessed with at least 4 small abscesses or at least 1 abscess with a diameter greater than 2.5 cm.

### Chemical analyses

Diets and orts were sampled weekly, oven dried at 55 °C for 72 h and composited by weight period. For further analysis, the composited samples were ground through a 1-mm screen (Wiley mill; Philadelphia, PA) and dried at 135 °C for 2 h to determine analytical DM (AOAC, 2005; method 930.15). The organic matter (**OM**) content was calculated as the difference between 100 and percentage of ash that was measured at 500 °C for 5 h (AOAC, 2005; method 942.05). NDF was determined as described by Van Soest et al. (1991) using heat-stable α-amylase and sodium sulfite. Acid detergent fiber was determined according to AOAC (AOAC, 2005; method 973.18). The NDF and ADF values were expressed inclusive of residual ash. Starch content was determined by enzymatic hydrolysis of α-linked glucose polymers as described by Rode et al. (1999). Nitrogen (N) content was analyzed from ball-ground subsamples (Retsch MM 400; Retsch Inc., Newtown, PA) by flash combustion with thermal conductivity detection (Carlo Erba Instruments, Milan, Italy). Total N content was expressed on crude protein (CP)-basis (N × 6.25). Ruminal VFA were quantified using a gas chromatograph (model 5890, Hewlett-Packard Lab, Palo Alto, CA) equipped with a capillary column (30 m × 0.32 mm i.d., 1-μm phase thickness, Zebron ZB-FAAP, Phenomenex, Torrance, CA) and flame ionization detection with helium as the carrier gas. The oven temperature was held at 170 °C for 4 min, which was then increased by 5 °C/min to 185 °C, and then by 3 °C/min to 220 °C, and held at this temperature for 1 min. The injector and detector temperatures were 225 °C and 250 °C, respectively. Concentrations of NH_3_-N in ruminal contents were determined as described by Rhine et al. (1998). Cortisol content of hair samples was analyzed as described previously Moya et al. (2013) using an immunoassay kit (DetectX Kit, Arbor Assays, Ann Arbor, MI).

### Statistical analysis

Data were analyzed using the Mixed procedure of SAS (Version 16.0.0, SAS Inst. Inc. Cary, NC) as a completely randomized block design. For the analysis of repeated measures, the best-fitting covariate structure (first order autoregressive) was selected based on the smallest Akaike’s information and Bayesian information criteria. Denominator degrees of freedom were determined using the Kenward–Roger option. The model used to analyze DMI and growth performance parameters over the entire length of the experiment included the fixed effects of grain processing, uNDF concentration, their interaction, and pen as a random effect. Days on feed were included as a repeated measure.

Individual steers within a pen were considered the experimental unit for eating behavior data. The model included fixed effects of grain processing, uNDF concentration, days on feed, and their 2- and 3-way interactions, as well as the random effect of steer within pen. Monthly measurement was used as a repeated measure. Denominator degrees of freedom were determined using the Kenward–Roger option. For analysis of ruminal pH and fermentation parameters, the same model was used, with data from day 0 included as a covariate.

The model for blood metabolites, acute phase proteins (**APP**), and hair cortisol included the fixed effects of grain processing, uNDF concentration, month, and 2- and 3-way interactions. Steer was included as a random effect, and month as a repeated measure using the MIXED procedure of SAS. To test treatment effects within each month, month and the interaction of treatment and month were removed as fixed effects. Data from day 0 were included in the model as covariates.

Carcass data were analyzed using the Mixed procedure of SAS with grain processing, uNDF concentration, and their interaction as fixed effects considering pen as a random effect. Meat quality grade and liver abscess data were analyzed using the GLIMMIX procedure (SAS, Version 9.1; SAS Institute, Inc. Cary, NC) with a binomial error structure and logit data transformation. Liver scores were expressed as a percent of steers with liver abscess incidence, or severe absence incidence. For all parameters, level of significance was declared at P < 0.05 with trends discussed at 0.05 ≤*P*≤ 0.10.

## Results

### Feed intake, growth performance, and eating behavior

There were no processing methods (dry- vs. steam-rolling) by dietary uNDF concentration (low vs. high) interactions for DMI, final BW, ADG, or G:F (Table 3). Neither processing method nor the dietary uNDF concentration affected DMI, growth performance, or feed efficiency. Regarding eating behavior, interactions between grain processing and uNDF concentration were insignificant for most variables, except for the standard deviation of DMI (*P* = 0.01), which was lower (*P* < 0.01) for the dry-rolled—low uNDF diet compared to all others. Overall, eating behavior was not affected by processing method, but steers fed the high uNDF diet spent more time at the feed bunk (min/d; *P* = 0.01) and had longer meal duration (min/meal; *P* = 0.01) than steers fed the low uNDF diet. In contrast, increased diet uNDF decreased the inter-meal interval (*P* = 0.04) and eating rate (*P* = 0.01).

**Table 3.** Effect of grain processing and dietary concentration of uNDF on growth performance and eating behavior of finishing feedlot steers

	Dry-rolled		Steam-rolled			*P*-value		
Item	Low^1^	High^1^	Low	High	SEM	Rolling	uNDF	Int^2^
Growth								
No of steers/pens^3^	94/8	94/8	94/8	94/8				
Initial BW, kg	439	441	439	442	3.5	0.96	0.45	0.95
Final BW, kg	722	719	733	722	4.8	0.17	0.16	0.38
DMI, kg/d	11.1	11.0	11.2	11.1	0.12	0.24	0.33	0.79
ADG, kg/d	2.03	2.01	2.11	2.04	0.035	0.13	0.22	0.49
G:F	0.184	0.183	0.187	0.184	0.003	0.48	0.45	0.66
Eating behavior^4^								
No of steers^5^	50	50	50	50				
DMI, kg/d	11.0	10.9	11.2	11.3	1.39	0.06	0.99	0.43
DMI-SD^6^	1.38^b^	1.54^a^	1.62^a^	1.56^a^	0.029	0.01	0.12	0.01
Bunk attendance, min/d	82.5	96.8	81.4	97.3	1.89	0.90	0.01	0.68
Meal frequency, meals/d	7.3	7.6	7.2	7.7	0.27	0.95	0.16	0.74
Duration, min/meal	12.0	14.0	12.1	13.2	0.51	0.51	0.01	0.33
Meal interval, min	206.7	198.1	207.6	186.3	7.11	0.45	0.04	0.38
Meal size, kg DM/meal	1.64	1.59	1.68	1.54	0.059	0.90	0.13	0.48
Eating rate, g DM/min	139.7	117.3	142.0	120.7	2.89	0.34	0.01	0.87

^1^Low = low concentration of undigestible NDF (uNDF) diet; High = high concentration of uNDF diet.

^2^Interaction between barley rolling method and dietary uNDF concentration.

^3^4 pens housed 30 steers per pen (1 pen/treatment), and 28 pens housed 10 steers per pen (7 pens/treatment).

^4^Distinct feeding events were selected based on a meal criterion of 300 s as described by (Schwartzkopf-Genswein et al., 2002).

^4^Eight pens housed 10 steers per pen (2 pens/treatment), and 4 pens housed 30 steers per pen (1 pen/treatment) were equipped with Growsafe systems.

^5^DMI variation (SD = standard deviation of DMI over a week).

^a,b^Least square means within a row with different superscripts differ (*P* < 0.05).

### Ruminal pH and fermentation characteristics

Maximum ruminal pH was not affected by uNDF content of the dry-rolled barley diet, but it was greater (*P* < 0.01) for the high compared to low uNDF—steam-rolled barley diet (interaction, *P* = 0.02; Table 4). Other ruminal pH parameters including mean and minimum pH, and the duration that pH was <6.0, <5.8, and <5.2 or the area under the curve at pH 6.0, 5.8, and 5.2, were not affected by the processing method × uNDF interaction. Overall, processing method did not affect ruminal pH; whereas, steers fed diets with high uNDF had an increased mean pH (*P* = 0.04) and reduced (*P* = 0.01) duration that pH was <6.0, 5.8, and 5.2. Feeding high uNDF diets to steers also tended to decrease the area that pH was <6.0 (*P* = 0.07) and 5.8 (*P* = 0.10).

**Table 4.** Effect of grain processing and dietary concentration of uNDF on ruminal pH and fermentation characteristics in finishing feedlot steers

	Dry-rolled		Steam-rolled			*P*-value		
Item	Low^1^	High^1^	Low	High	SEM	Rolling	uNDF	Int^4^
No. of steers	6	6	6	6				
Ruminal pH								
Mean	5.57	5.89	5.66	6.03	0.157	0.48	0.04	0.87
Minimum	4.62	5.22	5.15	5.38	0.264	0.21	0.13	0.50
Maximum	6.76^a^	6.69^a^	6.41^b^	6.74^a^	0.074	0.05	0.10	0.02
pH < 6.0, h/d	15.0	13.8	18.6	12.3	1.29	0.41	0.01	0.06
pH < 5.8, h/d	12.3	10.5	15.7	8.7	1.49	0.58	0.01	0.10
pH < 5.2, h/d	4.2	1.5	2.8	1.1	0.77	0.27	0.01	0.52
Area under curve								
pH 6.0, pH × h/d	16.1	7.6	9.6	4.9	3.47	0.20	0.07	0.58
pH 5.8, pH × h/d	12.7	4.5	6.1	2.8	3.31	0.23	0.10	0.47
pH 5.2, pH × h/d	6.9	0.2	0.3	0.1	3.14	0.30	0.29	0.31
VFA^2^								
Total, m*M*	107.7	114.1	116.7	107.3	4.20	0.81	0.72	0.08
Acetate, %	51.0	52.5	49.1	53.2	0.98	0.56	0.04	0.23
Propionate, %	37.6	36.5	39.4	35.9	1.36	0.68	0.13	0.41
Butyrate, %	6.5	6.2	7.2	7.1	0.52	0.14	0.78	0.85
BCVFA^3^, %	2.0	2.2	1.7	2.1	0.21	0.39	0.12	0.73
Valerate, %	2.5	2.4	2.4	1.6	0.27	0.16	0.11	0.22
Caproate, %	0.34	0.30	0.26	0.15	0.024	0.01	0.01	0.13
Acetate:propionate	1.37	1.49	1.30	1.59	0.099	0.90	0.06	0.40
Ammonia N, m*M*	13.7	14.9	12.5	13.2	0.95	0.14	0.35	0.77

^1^Low = low concentration of undigestible NDF (uNDF) diet; high = high concentration of uNDF diet.

^2^Molar percentage of total volatile fatty acids.

^3^Branched chained volatile fatty acids.

^4^Interaction between barley rolling method and dietary uNDF concentration.

^a,b^Least square means within a row with different superscripts differ (P < 0.05).

Total ruminal VFA concentrations and individual VFA molar proportions did not differ between dry- and steam-rolled barley diets, except for a lower (*P *= 0.01) proportion of caproate with steam- vs. dry-rolled barley (Table 4). The high uNDF diet resulted in a greater (*P* = 0.04) molar proportion of acetate and numerically lower (*P* = 0.13) molar proportion of propionate, resulting in a trend for the acetate to propionate ratio (*P* = 0.06) to be greater with high vs. low uNDF diets. There were no interactions (*P* ≥ 0.08) between processing method and uNDF content for concentrations of total VFA and NH_3_–N, or individual proportions of VFA.

### Blood metabolites, acute phase protein, and hair cortisol

There was an interaction between processing method and uNDF content for blood glucose concentration on day 112 (*P* = 0.01) and overall (*P* = 0.02); where, high uNDF vs. low uNDF steers had lower glucose concentrations on day 112 (*P* < 0.01) and overall (*P* = 0.03) when fed the dry-rolled barley diet, whereas it was greater on day 112 (*P* = 0.02) when fed steam-rolled barley (Table 5). Blood insulin concentrations were greater either with steam- than dry-rolled barley (*P* = 0.01) or with high uNDF relative to low uNDF (*P* = 0.04) on day 56, whereas the insulin measured across sampling days was not affected (*P* = 0.19) by treatment. For blood APP, the Hp concentration was neither affected by processing method nor by diet uNDF content. The LBP concentration on day 56 was greater (*P* < 0.01) in steam- compared to dry-rolled barley fed steers and on day 112 and overall there was a processing method and uNDF content interaction, where it decreased (*P* = 0.05) in the high uNDF—dry-rolled barley diet, but increased (*P* = 0.02) in the high uNDF—steam-rolled barley diet. There was no difference in LBP concentration between low and high uNDF. The SAA concentration did not differ on day 112, but it was greater on day 56 (*P* = 0.05) and overall (*P* = 0.04) for steam- vs. dry-rolled barley diets. The concentration of SAA was not affected (*P* ≥ 0.32) by dietary uNDF content. Hair cortisol concentrations did not differ (*P* ≥ 0.26) among treatments.

**Table 5.** Effect of grain processing and dietary concentration of uNDF on blood metabolites, acute phase protein and hair cortisol concentration in finishing feedlot steers

	Dry-rolled		Steam-rolled			*P*-value		
Item^1^	Low^2^	High^2^	Low	High	SEM	Rolling	uNDF	Int^3^
No of steers	20	20	20	20				
Glucose, mg/dL								
d0	78.2	66.5	100.7	93.0	3.06	0.01	0.15	0.31
d56	88.7	87.1	82.8	77.3	2.95	0.04	0.18	0.44
d112	69.1^a^	53.8^bc^	45.7^c^	59.2^ab^	4.64	0.14	0.81	0.01
Overall	78.8^a^	70.3^b^	64.4^b^	68.4^b^	3.08	0.04	0.41	0.02
Insulin, ng/mL								
d0	4.80	4.59	7.06	5.14	1.59	0.38	0.51	0.59
d56	2.51	3.73	4.17	5.39	0.57	0.01	0.04	0.99
d112	3.99	7.22	7.64	2.78	1.92	0.85	0.66	0.06
Overall	3.23	4.38	5.91	4.87	0.99	0.18	0.78	0.19
Hp, mg/mL								
d0	1.10	1.09	0.65	0.69	0.160	0.01	0.94	0.86
d56	0.50	0.52	0.64	0.71	0.047	0.01	0.38	0.59
d112	0.88	1.00	0.92	0.85	0.079	0.52	0.76	0.26
Overall	0.69	0.76	0.78	0.78	0.046	0.26	0.49	0.48
LBP, mg/mL								
d0	44.07	54.40	58.51	53.04	9.41	0.49	0.80	0.41
d56	37.68	36.37	52.61	63.78	5.61	0.01	0.35	0.29
d112	50.21^a^	29.67^bc^	22.91^c^	35.42^ab^	4.56	0.03	0.75	0.01
Overall	43.26^ab^	32.76^c^	37.76^bc^	50.10^a^	3.74	0.12	0.81	0.01
SAA, µg/mL								
d0	45.45	42.91	35.64	30.99	6.84	0.12	0.60	0.88
d56	30.03	22.61	34.54	35.05	4.23	0.05	0.42	0.35
d112	35.65	40.62	54.23	39.99	5.54	0.12	0.42	0.09
Overall	32.76	31.61	44.38	37.52	3.98	0.04	0.32	0.48
Cortisol, pg/mg								
d0	17.68	12.59	6.51	5.23	2.13	0.01	0.14	0.37
d56	10.48	13.69	14.28	14.21	2.35	0.41	0.51	0.49
d112	9.90	12.07	11.75	11.65	1.36	0.64	0.43	0.41
Overall	10.28	13.17	12.88	12.62	1.41	0.51	0.36	0.26

^1^HP = haptoglobin; LBP = lipopolysaccharide-binding protein; SAA = serum amyloid-A.

^2^Low = low concentration of undigestible NDF (uNDF) diet; High = high concentration of uNDF diet.

^3^Interaction between barley rolling method and dietary uNDF concentration.

^a,b,c^Least square means within a row without a common superscript letter are different (P < 0.05).

### Carcass traits and liver score

There was no interactions (*P* ≥ 013) between grain processing method and dietary uNDF concentration on carcass traits and liver abscesses (Table 6). The LM area (cm^2^) was greater (*P* = 0.01) and the proportion of carcasses graded as AAA was lower (*P* = 0.01) with steers fed steam-rolled barley than steers fed dry-rolled barley. Carcass traits were not affected (*P* ≥ 0.17) by increasing the dietary uNDF concentration. Liver scores were not affected (*P* ≥ 0.13) by processing method nor by dietary uNDF content.

**Table 6.** Effect of grain processing and dietary concentration of uNDF on carcass traits and liver scores of finishing feedlot steers

	Dry-rolled		Steam-rolled			*P*-value		
Item	Low^1^	High^1^	Low	High	SEM	Rolling	uNDF	Int^2^
No of steers/pen^3^	94/8	94/8	94/8	94/8				
Carcass traits								
HCW, kg	421	418	425	421	3.2	0.35	0.27	0.77
Dressing, %	58.2	57.9	58.0	58.1	0.15	0.90	0.50	0.17
Meat yield^4^, %	52.7	52.6	52.7	54.4	0.67	0.19	0.26	0.17
LM area, cm^2^	96.3	96.8	99.9	103.2	1.78	0.01	0.29	0.44
Back fat, mm	18.8	19.3	19.5	18.6	0.64	0.95	0.78	0.30
AAA^5^, %	87.8	76.9	71.3	68.4	0.09	0.01	0.17	0.39
Liver score, %								
Abscessed^6^	66.7	76.9	71.3	66.3	0.10	0.60	0.55	0.13
Severely^7^	46.7	39.6	44.7	38.9	0.17	0.84	0.23	0.87

^1^Low = low concentration of undigestible NDF (uNDF) diet; High = high concentration of uNDF diet.

^2^Interaction between barley rolling method and dietary uNDF concentration.

^3^Carcass data of 24 ruminally cannulated steers (6 steers per treatment) were not included.

^4^Estimated lean yield = 57.96 - 0.027 HCW + 0.202 LM area—0.703 Back fat.

^5^Canada grade AAA = equivalent to USDA Choice.

^6^The percentage of livers with at least 1 abscess.

^7^The percentage of livers with at least 4 small abscesses or at least 1 abscess with a diameter greater than 2.5 cm.

## Discussion

### Particle size distribution, peNDF, and uNDF

In the present study, barley-grain was the primary source of dietary uNDF (75 to 85 % of the total uNDF), whereas the difference in uNDF concentrations between the low and high uNDF diets resulted from the replacement of barley silage with barley straw due to the higher uNDF content of straw. Altering forage source or forage quality is a practical approach to manipulate dietary uNDF concentrations and it has been used in a number of studies with dairy cows (Cotanch et al., 2014; Kahyani et al., 2019) and feedlot cattle (Ran et al., 2021; Pereira et al., 2023b). Although this approach confounds forage type and uNDF concentration, it may have an advantage over increasing forage inclusion as a method to increase dietary uNDF. The difference in uNDF concentrations between low and high uNDF diets was small (only 0.9% points), but similar to our previous studies with finishing cattle (Ran et al., 2021; Pereira et al., 2023b). These studies revealed that even slightly elevated dietary uNDF concentrations can lead to increased chewing activity and improved ruminal pH (4.6% to 5.6% of diet DM; Ran et al., 2021). It also tended to increase the frequency of ruminal contractions and to improve the digestive tract barrier function of finishing beef heifers (7.1% vs. 8.5% DM; Pereira et al. 2023b).

The low and high uNDF diets had similar peNDF values to enable the impact of uNDF to be assessed independent of peNDF. Although the NDF concentration of straw was greater than that of the silage, there were fewer particles retained on the 4-mm sieve (i.e., less pef with straw than silage), thus the substitution of straw for silage did not significantly increase the peNDF concentration of the diet. Thus, the slightly greater peNDF with high vs. low uNDF diets was attributable to the greater NDF concentration of straw vs. silage. By definition, peNDF relies on particle length, whereas uNDF is primarily associated with the chemical characteristics of NDF. Therefore, the effect of replacing silage with straw in our study was primarily a result of an increase in uNDF rather than peNDF. Moreover, when the peNDF concentration was estimated based solely on the forage sources, it did not vary with barley processing method, whereas the dietary peNDF concentration was much greater with steam-rolled barley than dry-rolled barley. This is a reflection that over 90% of steam-rolled barley was retained on 4- and 3.35-mm sieves, whereas only 7% of dry-rolled barley was retained on these sieves. Pereira et al. (2023b) suggested that the value of dietary peNDF may be overestimated with increasing inclusion of barley-grain in the diet. Alternatively, in the study by Ran et al. (2021), the dietary peNDF was not impacted by increasing the PI of dry-rolled barley as the grain particles were not retained on the 19- and 8-mm sieves that were used to estimate peNDF. Therefore, caution should be used when determining the pef of diets containing grains and pelleted supplements as retained particles on the 4-mm sieve may inflate dietary peNDF estimates (NASEM, 2016).

### Feed intake and growth performance

There were no individual or combinational effects of the grain processing method and dietary uNDF content on feed intake or growth performance of steers fed high-grain diets. The present findings are consistent with several reports that DMI, ADG, and feed efficiency of beef cattle-fed high-grain diets were similar for steam-rolled vs. dry-rolled barley (Owens et al., 1997; Dehghan-banadaky et al., 2007). Alternatively, our results are inconsistent with the observation of Nixdorff et al. (2020) where finishing steers fed either coarsely or moderately steam-rolled barley vs. dry-rolled barley exhibited decreased DMI and improved feed efficiency. The discrepancy between the 2 studies is likely due to differences in the degree of processing. Nixdorff et al. (2020) processed to a PI of 67% for coarse, and 54% for moderate steam-rolled barley compared to a PI of 77 % for dry-rolled barley. In contrast, the current study processed both the dry- and steam-rolled barley to a PI of 70%. The more aggressive PI of steam-rolled barley may explain the improved feed efficiency as a result of reduced DMI and increased starch digestibility observed by Nixdorff et al. (2020). A decrease in PI linearly increased the in situ ruminal starch digestibility of steam- (Yang et al., 2000) and dry-rolled barley (Beauchemin et al., 2001). In the present study, the lack of processing method effect on growth performance suggests that starch digestion in the rumen did not differ between cattle-fed dry- and steam-rolled barley. This is supported by the similar individual and total VFA concentrations observed between dry- and steam-rolled barley-grain and minimal changes in ruminal pH.

The lack of an effect of uNDF concentration on DMI and growth performance in the present study is in agreement with Pereira et al. (2021) who did not observe any changes in DMI, ADG, or G:F of finishing steers when dietary uNDF was increased by substituting barley-silage with barley-straw. The authors speculated that the lack of response could be due to small changes in dietary uNDF intake (from 0.15% to 0.17% of BW) and that uNDF intake does not regulate DMI in finishing cattle-fed barley-based diets. The DMI of finishing steers was also unaffected by increasing dietary uNDF content in several other studies (Ran et al., 2021; Pereira et al., 2023a, 2023b), where differences in dietary uNDF were also small (4.6% to 5.6% or 7.1% to 8.5%). It has been reported that uNDF is a better predictor of DMI in dairy cattle than lignin (Raffrenato et al., 2019). Pereira et al. (2023a) reported no differences in DMI between heifers fed finishing diets with similar uNDF contents containing either 10% pelleted or 10% chopped straw, even though this resulted in a substantial difference in peNDF content. The authors (Pereira et al., 2023a) also found no difference in the ruminal uNDF pool (1.72 to 1.93 kg), even though the intake of uNDF differed (0.52 to 0.75 kg/d) among treatments, possibly because the uNDF associated with barley-grain is retained in the rumen longer. Similarly, Cotanch et al. (2014) reported reduced DMI with higher uNDF in the diet, with the intake of uNDF being limited at 2.0 to 2.6 kg/d. It was further noticed that the ratio of the ruminal uNDF pool to uNDF intake in dairy cows was approximately 1.6 across a range of diets that contained corn silage and hay crop silage. In finishing feedlot diets, the low concentration of forage inclusion may result in uNDF failing to reach concentrations in the rumen that regulate DMI. Furthermore, the lack of dietary uNDF impact on ADG and G:F is consistent with its effect on total ruminal VFA concentration and ruminal fermentation efficiency (i.e., acetate to propionate ratio).

### Eating behavior

The feeding behavior in the present study appeared to be different from a previous study even though similar barley-based finishing diets were fed (Brand et al., 2019). For example, in the current study, the duration of bunk attendance and meal frequency were less, but mean DMI, eating rate, and meal size were greater than those reported by Brand et al. (2019). Schwartzkopf-Genswein et al. (2002, 2011) reported existing correlations between feeding behavior and growth performance, but it was suggested that this relationship may vary with season, breed, sex, source, feeding management, diets, and stress levels. Studies investigating the impact of barley processing methods on eating behavior are scarce. Soltani et al. (2009) reported no difference in eating time, expressed either on a daily or per kilogram of DMI basis between dairy cows fed ground- or steam-rolled barley-grain. This is in agreement with the present study where eating behavior was similar among steers fed dry- vs. steam-rolled barley. Lack of an impact of processing method on eating behavior is consistent with similar DMI and growth performance among steers. Schwartzkopf-Genswein et al. (2011) reported that high ADG cattle spent more time at the bunk, and had greater eating rates, but attended the bunk less frequently than the low ADG cattle. A positive correlation (*r* = 0.38) between ADG and bunk attendance duration in feedlot steers has also been identified (Schwartzkopf-Genswein et al., 2002).

Changes in eating behavior with differing uNDF content did not affect growth performance. The lack of association between eating behavior and growth performance could be due to the similar DMI in the present study. In fact, studies that identified relationships between feeding behavior and ADG or G:F reported differences in DMI among treatments (Schwartzkopf-Genswein et al., 2002, 2011; Nkrumah et al., 2006). The greater bunk attendance duration and meal length with high uNDF concentration in the current study is consistent with several other studies with finishing beef cattle-fed barley-based diets. Ran et al. (2021) reported that increasing uNDF concentration in finishing feedlot diets increased eating time (min/d and min/kg DMI) without affecting ruminating time. Pereira et al. (2023a) found that an increase in diet uNDF concentration without a concurrent increase in peNDF, increased eating time (min/kg DM) and number of meals. Ran et al. (2021) proposed that the increased eating time associated with higher uNDF diets was likely due to sorting of longer particles and the lower palatability of straw as compared to silage. Greater sorting behavior was observed for dairy cows consuming feed at a slower rate (Greter and Devries, 2011). Additionally, an increase in the duration of bunk attendance and meal length with increasing uNDF in the present study may be linked to a small increase in diet DM (from 84.5 to 91.5%, on average) as straw replaced silage.

### Ruminal pH and fermentation characteristics

Ruminal pH did not differ between dry- and steam-rolled barley, which is consistent with the findings of similar total ruminal VFA concentrations across treatments. Numerous in vitro and in situ studies have compared impact of processing methods on ruminal digestion of barley-grain. Some studies have observed a reduction in the rate of in situ degradation of protein and starch in steam- vs. dry-rolled barley (Fiems et al., 1990; Engstrom et al., 1992; Tothi et al., 2003). It has been proposed that increased heat and moisture during steam-rolling strengthens protein–starch bonds within the endosperm of barley (a, 2003b); however, steam can also gelatinize starch, making it more degradable than starch in dry-rolled barley (Mathison et al., 1991). A previous study in our lab found that particle size was the primary predictor of the rate of DM disappearance of barley-grain in the rumen (Zhao et al., 2015). In the present study, more particles passed through the 1.18-mm sieve with dry-rolled barley (10%) than steam-rolled barley (2%), a factor that may have offset the higher degradation rate of gelatinized starch in steam-rolled barley.

Increased uNDF concentration in finishing diets increased the mean ruminal pH and reduced the duration that pH was below 6.0, 5.8, and 5.2. These results are consistent with previous studies where increasing uNDF concentrations by replacing silage with straw in finishing diets decreased the duration that pH was below 5.6 (Ran et al., 2021) and 5.5 (Pereira et al., 2023a). These studies also observed that increased diet uNDF increased the number of meals, eating and ruminating time, while eating rate, time between ruminal contractions, ruminal VFA concentrations, and total tract digestibility of OM were decreased; responses that were associated with an increase in ruminal pH. Similarly, in the present study, the increase in ruminal pH with uNDF was associated with changes in eating behavior including increased duration of bunk attendance, decreased inter-meal intervals, and eating rate. The increase in the proportion of acetate and the acetate to propionate ratio with higher uNDF in the diet suggests that the effects of diet uNDF on ruminal fermentation were detectable even when the uNDF content of finishing diets was increased from 5.7% to 6.6%.

### Blood metabolites, acute phase protein, and hair cortisol

Blood glucose is an important indicator of energy status and is related to the amount of ingested starch (Walker and Harmon, 1995; Oba and Allen, 2003; Liu et al., 2010). The energy requirement of finishing steers was met in the current study (NASEM, 2016), thus the reduction in plasma glucose concentrations associated with steam-rolled barley on days 56 and 112 (with low uNDF) and by high uNDF on day 112 with dry-rolled barley was unexpected. The reduced plasma glucose could be related to differences in the rate of liberation of glucose from starch in dry- vs. steam-rolled barley, but given the high fermentation of starch with both processing methods it seems more likely that they arise as a result of an increase in blood APP (Ran et al., 2018). Blood insulin is normally released in response to high plasma glucose concentrations, but the opposite response may reflect an inflammatory response. The differences observed in blood glucose and insulin concentrations are inconsistent with the lack of difference among treatments in DMI, ADG, and ruminal propionate proportion (Oba and Allen, 2003; Liu et al., 2010; Rathert-Williams et al., 2021). The present results are also not in agreement with our previous studies using finishing cattle-fed diets with varying concentrations of grain processing and uNDF (Ran et al., 2021; Pereira et al., 2023a, b). Brown et al. (2000) reported that plasma insulin in steers fed corn differing in processing method or degree, varied with the sampling time within and between days. This suggests that more intensive sampling intervals would be required to evaluate an insulin response. In addition, changes in blood glucose and insulin may be related to inflammation as increased insulin was accompanied by increases in blood Hp, LBP and SAA on d56.

Haptoglobin, SAA, and LBP are APP and can bind bacterial endotoxins and are used to evaluate systemic responses to infection, inflammation, or trauma (Murata et al., 2004; Ceciliani et al., 2012). Feeding high amounts of rapidly fermentable carbohydrates decreases ruminal pH, alters microbial populations in the rumen, and increases concentrations of endotoxins in ruminal fluid (Andersen, 2003; Emmanuel et al., 2008). Blood endotoxins mainly originate from the rumen and hindgut and can induce systemic inflammatory responses (Plaizier et al., 2022). In the present study, blood concentrations of Hp, LBP and SAA changed even though ruminal pH and the duration that pH was <5.8 or <5.2 were not affected by processing methods. Similarly, Pereira et al. (2023a) reported no difference in ruminal pH, but lower plasma SAA and Hp concentrations were observed when 10% of the barley-grain in a finishing diet was replaced by pelleted straw.

Hair cortisol concentration has been used as biomarker of chronic stress in cattle (Schubach et al., 2020). The hair cortisol concentration in the hair of steers in the present study (12.2 pg/mL) was more than twice that of a study with beef heifers fed a high-concentrate diet (< 5 pg/mg; Moya et al., 2013; Rett et al., 2020), possibly reflecting increased stress; however, the lack of treatment effect indicated neither processing method or uNDF concentration altered hair cortisol concentrations. High-grain feeding (high risk of acidosis), weighing, and blood sampling throughout the experiment were all likely stressors for steers in the current experiment.

### Carcass traits and liver score

Lack of differences among treatments for carcass traits is consistent with the growth performance findings of this study and in agreement with previous reports that carcass characteristics are not strongly influenced by grain processing method (Bradshaw et al., 1996; Wang et al., 2003; Nixdorff et al., 2020). The cause of greater LM area with steam- than dry-rolling is unclear, and may lack relevance as there is no optimum LM size preferred by retail consumers (Sweeter et al., 2005). Moreover, reduction in the proportion of the AAA graded carcasses from steam-rolled barley fed steers is not consistent with previous studies assessing different grain processing methods. Pereira et al. (2021) reported that the greater proportion of AAA quality grade observed in cattle-fed barley-silage compared to wheat-silage was due to greater energy content of barley-silage. Studies evaluating effects of dietary uNDF concentration on carcass traits of beef cattle are limited in number. The current results are consistent with Pereira et al. (2021) who found that most carcass traits were not affected by uNDF, with the exception that the cattle-xfed barley-silage with low uNDF had greater dressing percentages than those fed high uNDF.

The method of barley-grain processing and diet uNDF concentration did not affect the proportion of abscessed livers; however, the proportion of mildly and severely abscessed livers (70%) in the current study was much greater than Canadian industry standards (32%) based on the national beef quality audit conducted in 2016 and 2017 (Canadian Cattle Association, 2018). The combination of low forage inclusion (6%) and aggressive barley processing (PI = 70%), and absence of any liver abscess control agents in the diet may explain the high incidence of liver abscesses. Although ruminal pH status was improved by elevating uNDF, the mean ruminal pH (5.79), and the duration that pH was below 5.8 (11.8 h per 24 h) were still high, with steers experiencing subclinical ruminal acidosis. Plaizier et al. (2008) characterized subacute ruminal acidosis as the duration of ruminal pH < 5.6 exceeding 180 min daily. McAllister et al. (1990) reported that rapid starch degradation in the rumen of steers fed high-grain diets can lower ruminal pH and cause digestive disturbances such as acidosis and rumenitis, increasing the incidence of liver abscesses.

In conclusion, only a few interactions between the grain processing method and uNDF content were detected for the variables measured, indicating that both dietary factors acted independently. Grain processing did not affect feed intake, eating behavior, growth performance, or ruminal pH, or fermentation characteristics in finishing steers fed high barley-grain diets. This study demonstrated that processing barley-grain using steam-rolling did not enhance growth performance or feed efficiency, but it also did not increase the risk of ruminal acidosis. Increasing dietary uNDF concentration by replacing silage with straw altered eating behavior, resulting in longer bunk attendance and meal duration, with fewer inter-meal intervals and eating rate. The diet with high uNDF content resulted in an increase in mean ruminal pH, shortened the duration of pH below 5.8, and altered fermentation pattern with more acetate production, suggesting a possible improvement in ruminal fiber digestion. This study further confirms that the manipulation of diet uNDF content in feedlot finishing diets is an effective nutritional strategy to reduce the risk of ruminal acidosis, without negatively impacting growth performance, feed efficiency, inflammatory and stress responses, or carcass quality.

## Abbreviations:

ADF: acid detergent fiber
ADG: average daily gain
APP: acute phase proteins
BW: body weight
CP: crude protein
DM: dry matter
DMI: dry matter intake
G:F: gain:feed
Hp: haptoglobin
LBP: lipopolysaccharide-binding protein
LM: longissimus
N: nitrogen
NDF: neutral detergent fiber
OM: organic matter
pef: physical effectiveness factor
peNDF: physically effective fiber
PI: processing index
SAA: serum amyloid-A
TMR: total mixed rations
uNDF: undigestible neutral detergent fiber
VFA: , volatile fatty acid
